# Relationships between the antral follicle count, steroidogenesis, and secretion of follicle-stimulating hormone and anti-Müllerian hormone during follicular growth in cattle

**DOI:** 10.1186/s12958-019-0534-3

**Published:** 2019-11-05

**Authors:** Kenichiro Sakaguchi, Yojiro Yanagawa, Koji Yoshioka, Tomoko Suda, Seiji Katagiri, Masashi Nagano

**Affiliations:** 1 Laboratory of Theriogenology, Graduate School of Veterinary Medicine, Hokkaido University, Sapporo, Hokkaido 060-0818 Japan; 2 Research Fellow of the Japan Society for the Promotion of Science, Chiyoda-ku, Tokyo, 102-0083 Japan; 3 Laboratory of Theriogenology, Department of Clinical Sciences, Faculty of Veterinary Medicine, Hokkaido University, Sapporo, Hokkaido 060-0818 Japan; 4 National Institute of Animal Health, NARO, Tsukuba, Ibaraki 305-0856 Japan; 5 Present address: Laboratory of Animal Reproduction Department of Animal Science, School of Veterinary Medicine, Kitasato University, Aomori, 034-8628 Japan

**Keywords:** Anti-Müllerian hormone, Antral follicle count, Follicle stimulating hormone, In vitro growth, Steroidogenesis

## Abstract

**Background:**

The antral follicle count (AFC) in mammalian ovaries positively correlates with female fertility. To clarify the causes of differences in fertility between low and high AFC cows, we investigated follicular growth dynamics and hormone concentrations in plasma, follicular fluid, and in vitro growth (IVG) media at different stages of follicular growth.

**Methods:**

Seven cows were divided into high AFC (*n* = 4, > 30 follicles) and low AFC (*n* = 3, < 30 follicles) groups based on the peak AFC detected by ultrasonography. These cows were subjected to estrous synchronization, daily ovarian ultrasonography, and blood collection. Their follicular fluid was collected from dominant follicles at different stages (selection, luteal, and ovulatory phases). In another experiment, we cultured oocyte-cumulus-granulosa cell complexes collected from early antral follicles (< 1 mm) for 12 days. Estradiol-17β (E_2_), testosterone (T), progesterone (P_4_), and anti-Müllerian hormone (AMH) concentrations in follicular fluids and plasma were measured. Plasma follicle-stimulating hormone (FSH) concentrations were examined. E_2_, P_4_, and AMH concentrations were also measured in IVG media.

**Results:**

The numbers of small (< 4 mm) and intermediate (4–8 mm) follicles were larger in the high AFC group than in the low AFC group (*P* < 0.05). The number of intermediate follicles was stable in the low AFC group, indicating consistent development. However, the number of these follicles fluctuated in the high AFC group. Plasma FSH concentrations were higher, whereas E_2_ and T concentrations were lower in the low AFC group (*P* < 0.05). E_2_ concentrations and the E_2_/P_4_ ratio in ovulatory follicles and IVG media on day 8 were higher in the high AFC group (*P* < 0.05). AMH concentrations in plasma and IVG media (*P* < 0.01) were higher in the high AFC group.

**Conclusions:**

The weaker response to FSH of granulosa cells caused low E_2_ production in the low AFC group, resulting in high FSH concentrations and the consistent development of intermediate follicles. Conversely, higher E_2_ concentrations suppressed FSH secretion in the high AFC group. Granulosa cells in the high AFC group had the ability to produce more AMH than those in the low AFC group throughout IVG culture.

## Background

The primary roles of the ovaries are to support the growth and maturation of oocytes for the acquisition of fertilizability and competence for embryonic and fetal development, as well as the production of sex steroid hormones to induce the estrous cycle and sustain pregnancy. These ovarian functions are regulated by gonadotrophins and steroid hormones. In mono-ovulatory species, the emergence of follicular growth is induced by the surge-like secretion of follicle-stimulating hormone (FSH). A dominant follicle is then selected as the decrease in the level of FSH by the inhibitory effects of estradiol-17β (E_2_) and inhibin secreted by the follicles themselves. The dominant follicle continues to grow due to the stimulation by luteinizing hormone (LH), resulting in ovulation [1, 2]. Most follicles degenerate during follicular growth, and only a small proportion of follicles develop and ovulate [1, 2].

The ovarian reserve, the pool of primordial follicles in a pair of ovaries in individuals, is defined as the potential ability of ovarian function [3, 4] and is an indicator of female fertility in mono-ovulating species, such as humans [4] and cattle [5]. The peak number of antral follicles in a pair of ovaries during follicular waves counted by ultrasonography (the antral follicle count; AFC) positively correlates with the number of primordial follicles [6] and may be used to estimate the ovarian reserve [7]. Although AFC fluctuates during the estrous cycle and markedly varies between individuals, the peak AFC during the estrous cycle shows high repeatability in individual cattle [7]. Cattle with a high number of antral follicles in a pair of ovaries showed higher reproductive performance, such as higher fertility [8], a shorter open period [8], and higher responsiveness to superovulation [9], than cattle with a low number of antral follicles, even though they were in the same age class. We previously reported that the fertilizability of oocytes after in vitro fertilization (IVF) collected from cattle by ultrasound-guided ovum-pick up (OPU) was higher in high AFC cows having 30 or more antral follicles in a pair of ovaries at the time of OPU than in low AFC cows having less than 30 antral follicles at a 3- or 4-day interval of OPU [10]. In contrast, when we extended the interval of OPU to 7 days, the fertilizability of oocytes in high AFC cows was impaired and became less than that in low AFC cows, whereas the fertilizability of oocytes derived from low AFC cows was similar regardless of the OPU interval [10]. These findings indicate that the growth dynamics of antral follicles differ between high and low AFC cows, and the degeneration of antral follicles at the selection phase in the follicular wave may occur earlier in high AFC cows than in low AFC cows. However, the reason for this reversal in the relationship between AFC and oocyte fertilizability remains unclear. Furthermore, we conducted an in vitro growth (IVG) culture of bovine oocyte-cumulus-granulosa complexes (OCGCs) [11, 12], which enables bovine oocytes without maturational competence from early antral follicles to grow to the stage acquiring competence for maturation and development to the blastocyst stage [13–15] and offspring [13, 14]. By using this technology, we investigated follicular function, the acquisition of oocyte competence, and steroidogenesis in granulosa cells, and estimated follicular growth dynamics from the period during which follicles cannot be detected by ultrasonography in vivo to the period during which oocytes acquire developmental competence in high and low AFC cows. Consequently, OCGCs derived from early antral follicles (0.5–1.0 mm in diameter) in the high AFC group having 25 or more antral follicles (≥2.0 mm in diameter) in an ovary collected at a slaughterhouse showed higher oocyte maturational competence and fertilizability than those in the low AFC group having less than 25 antral follicles [11, 12]. Although the proliferation of granulosa cells was the same in both groups, E_2_ production by OCGCs was higher in the high AFC group than in the low AFC group [12]. We also revealed that granulosa cells surrounding in vitro-grown oocytes having higher maturational competence secreted more E_2_ and less progesterone (P_4_) than those surrounding less competent in vitro-grown oocytes using medium containing androstenedione (A_4_) instead of E_2_ [16].

Anti-Müllerian hormone (AMH) is a member of the transforming growth factor-β family. AMH is known to be a marker of ovarian reserve, and there is a strong correlation between AFC and AMH in human [17, 18] and cattle [19, 20]. AMH is secreted by the granulosa cells of primary to early antral follicles [17]. Some researchers conducted comparative studies of predictive values for human ART between AFC and blood AMH level, and suggested that predictive values of AFC and AMH were similar [4, 21–27]. In addition, some studies indicated that the predictive value of AFC was higher than that of AMH [28–30], although other studies demonstrated the contradictory results [18, 31, 32]. In AMH-deficient mice, the premature depletion of primordial follicles occurred [33], and AMH inhibited the activation of primordial follicles in cattle [34]. AMH inhibited the FSH-stimulated growth of antral follicles and E_2_ production by decreasing the sensitivity of preantral and antral follicles to FSH in mice [35], humans [36, 37], and sheep [38]. These findings suggest that AMH is an important regulator of follicular activation, follicular growth, and steroidogenesis in growing follicles. Furthermore, the plasma concentration of AMH positively correlated with the number of primordial follicles and AFC in cattle [6] and humans [17]. In cattle, the concentration of AMH in the follicular fluid of antral follicles (≥3 mm in diameter) decreased during follicular growth [39, 40]. Granulosa cells derived from antral follicles (3–5 mm in diameter) produced more E_2_ and AMH in high AFC cows having 25 or more follicles in a pair of ovaries than in low AFC cows having 15 or fewer antral follicles regardless of the addition of FSH to the in vitro culture of granulosa cells [41]. In the follicular fluid of antral follicles (5–7 mm in diameter), immediately before the selection of dominant follicles, AMH concentrations were similar between high AFC heifers and low AFC heifers, while E_2_ concentrations were lower in high AFC heifers than in low AFC heifers [42]. On the other hand, E_2_ concentrations in the follicular fluid of ovulatory follicles (approximately 15 mm in diameter) were higher in high AFC heifers than in low AFC heifers [43]. These findings indicated that AMH regulates FSH-stimulated E_2_ production during follicular growth, and this regulation may differ between each follicular growth stage. However, there is currently no information on the relationship between AMH concentrations in follicles after selection (≥8 mm in diameter) or before recruitment (< 4 mm in diameter) and AFC. In the present study, we investigated the relationship between AFC, follicular growth dynamics, FSH concentrations in plasma and steroid hormones, and E_2_, testosterone (T, one of the precursors of E_2_), and P_4_ concentrations in plasma and follicular fluid as the factors affecting oocyte developmental competence in high and low AFC cattle. We also investigated the relationship between AMH and AFC at follicular stages before recruitment by the IVG of OCGCs derived from early-antral follicles (< 1 mm in diameter) and ultrasound-guided follicular aspiration, respectively.

## Methods

### Animals

The present study was approved by the Institutional Animal Care and Use Committee of Hokkaido University. We selected experimental animals from non-pregnant Holstein cows kept at the experimental farm of Hokkaido University (*n* = 14; 6 lactating and 8 non-lactating cows). To exclude the negative impact of the postpartum negative energy balance on follicular development [44], we excluded the cows in early postpartum period. In addition, before starting the experiment, we examined ovaries using the ultrasound imaging device equipped with a 7.5 MHz rectal linear transducer (HLV-575 M; Honda Electronics) at a 12 days interval to select cows used for experiments from 14 non-pregnant cows (8 non-lactating and 6 lactating). Moving images of ultrasonography were saved into a video recorder (VR570; Toshiba Teli, Tokyo, Japan). We analyzed those images and removed cows with intermediate AFCs, ovarian cysts, and uterine disorder. As a result, we selected 3 low AFC cows (*n* = 3; 1 lactating and 2 non-lactating cows) and 4 high AFC cows (*n* = 4; 2 lactating and 2 non-lactating cows) for the experiment. Their age and parity were 9.0 ± 4.7 (mean ± SD) and 4.0 ± 2.2, respectively. Days after parturition in lactating cows at the start of experiments (day 0) were between 103 and 106.

### Chemicals

All chemicals used in the present study were purchased from Sigma-Aldrich (St. Louis, MO, USA) unless otherwise stated.

### Follicular fluid and blood collection and ultrasound examination

**Fig. 1:**
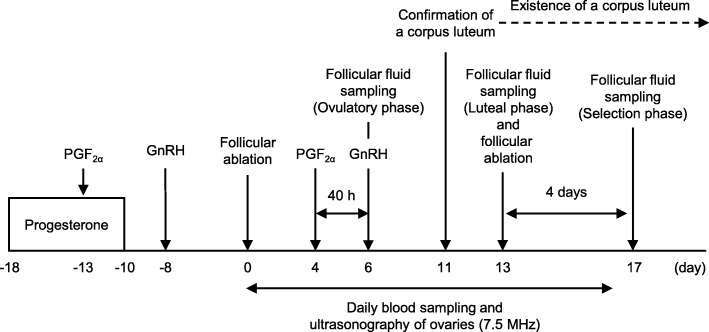
Schematic of the experimental design. The estrous cycles and follicular waves of cows were synchronized using hormonal treatments and follicular ablation between days − 18 and 0 [45]. On day 4, PGF_2α_ was injected to induce estrus. After 40 h, a dominant follicle just before the LH surge was aspirated and collected follicular fluid was defined as the ovulatory phase (day 6). Soon after follicular aspiration, GnRH was injected to induce luteinization of the dominant follicle. After 7 days, a dominant follicle growing with a corpus luteum was aspirated and collected follicular fluid was defined as the luteal phase (day 13). All visible follicles were then ablated. Four days later, the largest follicle was aspirated, and collected follicular fluid was defined as the selection phase (day 17). In three cows (1 low AFC and 2 high AFC), follicular ablation was performed again on day 16 and follicular fluid was collected on day 20. During days 0 to 16, we collected blood samples and monitored ovaries by ultrasonography daily

A schematic drawing of the ultrasound-guided follicular aspiration schedule is shown in Fig. 1. Estrous cycles and follicular waves in cows were synchronized for the collection of follicular fluid from follicles just before the expected time of the LH surge, as previously described [45]. Briefly, cows were inserted an intravaginal P_4_ device (1.9 g, CIDR 1900; Zoetis Japan, Tokyo, Japan) (day − 18). Five days after insertion of the P_4_ device, prostaglandin F_2α_ (PGF_2α_, 25 mg, Pronalgon F containing 5 mg/mL of dinoprost; Zoetis Japan) was injected intramuscularly (i.m.) (day − 13). The P_4_ device was removed 8 days after its insertion (day − 10). Two days later, a gonadotropin-releasing hormone (GnRH) analogue (200 μg, Conceral injection containing 50 μg/mL fertirelin acetate; Intervet, Osaka, Japan) was injected i.m. (day − 8). After 8 days, large follicles were ablated under an ultrasound imaging device (HS-2100; Honda Electronics, Aichi, Japan) equipped with a 9.0 MHz long-handled convex transducer (HCV-4710MV; Honda Electronics) for synchronization of the emergence of the follicular wave [46] (day 0). Follicles were aspirated using a single-lumen needle (17-gauge, 490 mm long; Misawa Medical, Ibaraki, Japan) connected to a 50-mL tube (Falcon 2070; Becton Dickinson, Franklin Lakes, NJ, USA) via a silicone tube (100 cm long, internal diameter of 1 mm). Four days later, PGF_2α_ was injected i.m. (day 4). Forty hours after the PGF_2α_ injection, the follicular fluid of the dominant follicle (ovulatory phase) was collected under ultrasonography (day 6). Regarding the collection of follicular fluid, a single-lumen needle was connected to a 5- or 10-mL syringe. Two cows had a large subordinate follicle (≥8 mm in diameter) after the collection of follicular fluid from the dominant follicle, and these follicles were also ablated. GnRH was then injected i.m. to induce a LH surge. Five days after the GnRH injection, the formation of a corpus luteum was confirmed by ultrasonography in all cows as previously described [47, 48] (day 11), and 2 days later, follicular fluid was collected from the dominant follicle (luteal phase) (day 13). All visible follicles were then ablated. Four days later, follicular fluid was collected from the largest follicle (selection phase) (day 17). One cow had 2 large follicles (9.6 and 8.7 mm) and we were unable to distinguish the dominant follicle that expressed LH receptors [49] under ultrasonography; therefore, we collected follicular fluid from these follicles and pooled it as one sample. In three cows (1 low AFC and 2 high AFC), follicular ablation was performed again on day 16 and follicular fluid was collected from the largest follicle on day 20 for collecting the follicular fluid at selection phase. During days 0 to 16, we examined ovaries daily using the ultrasound imaging device equipped with a 7.5 MHz rectal linear transducer and moving images of ultrasonography were saved into a video recorder. In all cows, corpus luteum was confirmed by the ultrasonography at the day of final follicular aspiration (days 17 or 20). We also collected blood daily by jugular or caudal venipuncture using ethylenediaminetetraacetic acid-loaded vacuum tubes for hormone measurements. Each tube was centrifuged at 3000 rpm at 4 °C for 10 min. Plasma samples were stored at − 30 °C until hormone assays were conducted. We also performed ovarian ultrasonography and blood collection on the days of hormone treatments, ablation of follicles, and sampling of follicular fluid. In the analysis of follicular growth dynamics, recorded moving images were subjected to frame-by-frame playback using a media player (Windows Media Player; Microsoft, WA, USA). The number of antral follicles was counted, and the diameter of each antral follicle was measured using digital caliper software (Hakarundesu; Onegland.net, Shizuoka, Japan). Antral follicles were divided into 3 categories according to their diameters (small: < 4 mm, intermediate: 4–8 mm, and large: ≥8 mm) because follicles of 4 mm or larger in diameter are generally considered to represent the emergence of follicles [50], while follicles of 8 mm or larger in diameter start to express LH receptors [49].

### Collection of OCGCs and the IVG culture

The ovaries of Holstein cows obtained from a local abattoir were stored in plastic bags at 20 °C and transported to the laboratory within 6–10 h of their collection. After the ovaries had been washed three times with physiological saline, slices of ovarian cortex tissues (thickness < 1 mm) were prepared using a surgical blade (no. 11) and stored in tissue culture medium 199 (TCM-199; Thermo Fisher Scientific, Roskilde, Denmark) supplemented with 0.1% polyvinyl alcohol, 25 mM 2-[4-(2-Hydroxyethyl)-1-piperazinyl] ethanesulfonic acid (HEPES), 10 mM sodium bicarbonate, and 50 μg/mL gentamicin sulfate (isolation medium, pH 7.4) at 37 °C, as described elsewhere [51]. Under a stereomicroscope, early antral follicles (0.5–1.0 mm in diameter) were dissected from sliced ovarian tissues using a surgical blade (no. 20) and fine forceps in a 90-mm petri dish that had a 1-mm scale on its bottom (FLAT, Chiba, Japan). OCGCs were isolated from early antral follicles using a pair of fine forceps and subjected to IVG as previously described [16]. Growth medium was HEPES-buffered TCM-199 supplemented with 0.91 mM sodium pyruvate, 5% (v/v) fetal calf serum (Invitrogen), 4 mM hypoxanthine, 4% (w/v) polyvinylpyrrolidone (MW 360,000), 50 μg/mL ascorbic acid 2-glucoside (Wako Pure Chemical Industries, Osaka, Japan), 55 μg/mL cysteine, 50 μg/mL gentamicin sulfate, and 10 ng/mL A_4_ as a precursor for E_2_. OCGCs with oocytes surrounded by a cumulus investment and attached mural granulosa-cell layer were cultured individually in a 96-well culture plate (Primaria 353,872; Corning Life Sciences, Tewksbury, MA, USA) with 200 μL of growth medium at 39 °C for 12 days in humidified air with 5% CO_2_. Every 4 days of the IVG culture, the viability of OCGCs was assessed by their morphological appearance [16]. OCGCs having an evenly granulated ooplasm that was completely enclosed by several layers of a healthy cumulus and granulosa cells were defined as surviving. OCGCs having oocytes with an abnormal appearance and/or denuded by a scattering cumulus and granulosa cells were defined as dead. Simultaneously, half (100 μL) of the growth medium of surviving OCGCs was replaced with the same amount of fresh medium. The spent media of surviving OCGCs collected on days 4, 8, and 12 of the culture were stored at − 30 °C until assays of steroid hormones and AMH.

### E_2_, P_4_, and T assays

E_2_, T, and P_4_ concentrations were measured using competitive double-antibody enzyme immunoassays. Steroid hormones in plasma samples were extracted as described previously with slight modifications for the T assay [52]. In the E_2_ assay, 2 mL of plasma was extracted with 6 mL of diethyl ether (Kanto Chemical, Tokyo, Japan). In the T assay, 1 mL of plasma was extracted with 3 mL of diethyl ether. In the P_4_ assay, 200 μL of plasma was extracted with 2 mL of diethyl ether. Diethyl ether was then decanted into a new tube after freezing the plasma. After evaporating diethyl ether, 0.5 mL of acetonitrile (Kanto Chemical) and 1 mL of hexane (Kanto chemical) were added and mixed well in the extracted samples for the E_2_ and T assays for delipidation. Thereafter, 1 mL of hexane was added, and hexane was discarded using an aspirator. Acetonitrile was evaporated after repeating delipidation by hexane three times. Samples were reconstituted with 100 μL (E_2_) or 200 μL (T) of assay buffer (145 mM NaCl, 40 mM Na_2_HPO_4_, and 0.1% bovine serum albumin (BSA) (w/v), pH 7.2). Extracted samples for P_4_ were reconstituted with 200 μL of assay buffer without delipidation using acetonitrile and hexane. Follicular fluid samples and spent media were assayed without extraction. Samples were diluted with assay buffer. Extracted samples from plasma were assayed without dilution or subjected to a 10-fold dilution. Follicular fluid was subjected to a 100- or 1000-fold dilution. Spent media were subjected to 2- to 2000-fold serial dilutions. After dilution, samples (20 μL) were incubated with 100 μL (E_2_ and P_4_) or 50 μL (T) of the primary antisera and horseradish peroxidase-labeled hormone in the wells of a 96-well microplate (Costar 3590; Corning, NY, USA) coated with the secondary antiserum at 4 °C for 16–18 h. The primary antisera used for the E_2_, T, and P_4_ assays were anti-estradiol-17β-6-carboxymethyloxime (CMO)-BSA (FKA204; Cosmo Bio, Tokyo, Japan), anti-testosterone-3-CMO-BSA (FKA102; Cosmo Bio), and anti-progesterone-3-CMO-BSA (KZ-HS-P13; Cosmo Bio), respectively. Goat anti-rabbit serum (111–005-003; Jackson Immuno Research, West Grave, PA, USA) was used as the secondary antiserum. After the washing of all wells four times with 300 μL of washing buffer (0.05% Tween 80), 150 μL of 3,3′,5,5′-tetramethylbenzidine (TMB) solution (5 mM citric acid, 50 mM Na_2_HPO_4_, 500 mM urea hydrogen peroxide, 1 mM TMB, and 2% dimethyl sulfoxide) was added to each well and incubated at 37 °C for 40 min. The absorbance of the solution in the wells was measured at 450 nm using a microplate reader (Model 550; Bio-Rad Laboratories, Tokyo, Japan) after stopping the chromogenic reaction with 50 μL of 4 N H_2_SO_4_. All samples were assayed in triplicate. Assay sensitivities were 0.049 pg/well for E_2_, 0.195 pg/well for T, and 0.391 pg/well for P_4_. The inter- and intra-assay coefficients of variations were 15.1 and 4.0% for E_2_, 7.1 and 7.4% for T, and 14.9 and 3.9% for P_4_, respectively.

### FSH and AMH assays

FSH plasma concentrations were measured using a competitive double-antibody time-resolved fluoroimmunoassay with Eu-labeled FSH as a probe with slight modifications [53]. A bovine FSH immunoassay kit consisting of bovine FSH antisera (AFP7722291), bovine FSH (iodination grade, AFP-9294C), and a reference standard of bovine FSH (AFP-5346D) was provided by the National Institute of Diabetes and Digestive and Kidney Diseases (NIDDK) National Hormone and Pituitary Program (NHPP) (Dr. A.F. Parlow, NHPP, Torrance, CA, USA). We mixed 10 μL of bovine FSH solution (500 μg/mL) with Eu-labeling reagent (PerkinElmer, Waltham, MA, USA), and incubated samples at 37 °C overnight according to the manufacturer’s instructions. Eu-labeled FSH was separated from free Eu by gel filtration with a column (inner diameter of 1.5 cm, 12.0 cm, Econo-Pac column; Bio-Rad Laboratories) of Sephadex G-50 (GE Healthcare, Chicago, IL, USA). Bovine FSH antisera and the reference standard of bovine FSH were diluted using assay buffer (PerkinElmer) containing 0.1% gelatin. Bovine FSH antisera (100 μL) were incubated in the wells of a 96-well microplate (FluoroNunc Modules; Nalge Nunc International, Rochester, NY, USA) coated with the secondary antiserum at 34 °C overnight. Goat anti-rabbit IgG (AP132; Merck Millipore, Burlington, MA, USA) was used as the secondary antibody. After the washing of all wells 10 times with 300 μL of washing buffer (0.1% (w/v) Tween 20, 150 mM NaCl, and 0.05% (w/v) NaN_3_ in 5 mM Tris buffer, pH 7.8), plasma samples without dilution (100 μL) were added to the wells and incubated at 34 °C overnight. After the incubation, wells were washed 12 times and Eu-labeled FSH was added to the wells, which were then incubated at 34 °C for 6 h. After the wells were washed 12 times, enhancement solution (100 μL, PerkinElmer) was added to each well and incubated at 34 °C for 5 min. The fluorescence of the solution in the wells was measured using a microplate reader (1420 ARVO_SX_ DELFIA; PerkinElmer). Assay sensitivity was 204.8 pg/mL for FSH. The inter- and intra-assay coefficients of variations were 17.2 and 13.3%, respectively.

AMH concentrations in plasma, follicular fluid, and spent media were measured using a commercial kit (Bovine AMH ELISA; Ansh Labs., Webster, TX, USA) according to the manufacturer’s instructions. Samples were diluted with a sample diluent in the kit. Follicular fluid was subjected to a 100- or 1000-fold dilution. Plasma samples were assayed without dilution or subjected to a 4-fold dilution. Spent media were subjected to 100-fold dilution. The absorbance of the solution in the wells was measured at 450 nm with a background wavelength correction at 630 nm using a microplate reader (iMark; Bio-Rad Laboratories, Tokyo, Japan). Assay sensitivity was 11.0 pg/mL for AMH. The inter- and intra-assay coefficients of variations were 4.3 and 2.5%, respectively.

### Experimental design

Cows were classified into the low AFC group (less than 30 follicles) and high AFC group (more than 30 follicles) based on the peak number of antral follicles (≥3 mm in diameter) in a pair of ovaries from days 0 to 16 as described in our previous study [10]. The number of small, intermediate, and large follicles from days 0 to 16 were compared between groups and days. In addition, the transition of the number of intermediate (4–8 mm) and large (≥8 mm) follicles from 1 to 6 days after follicular ablation and sampling on days 0 and 6, respectively, were examined. We also compared the number of antral follicles between groups and days after follicular ablation and sampling. FSH, E_2_, T, and P_4_ plasma concentrations from days 0 to 16 were compared between groups and days. These concentrations during the selection phase (2 to 4 days after follicular ablation on days 0 and 6) were compared between groups. AMH plasma concentrations on the representative date of each stage of follicular growth (selection phase; day 4, luteal phase; day 13, and ovulatory phase; day 6) were compared between groups and each stage of follicular growth. E_2_, T, P_4_, and AMH concentrations in follicular fluid samples were compared between groups and each stage of follicular growth. However, in an aspirating session for a cow of the collection of follicular fluid, follicular fluid was scattered in the line of the needle and the tube due to its small volume. In that case, to collect follicular fluid, we washed the line with Dulbecco’s phosphate-buffered saline without calcium or magnesium, and adjusted the total amount of collected solution to 10 mL (cm^3^). Hormone concentrations in follicular fluid were calculated based on the formula below.

Concentrations in follicular fluid (ng/mL) =

Concentrations in collected solution (ng/mL) × volume of the follicle (cm^3^)/10 (cm^3^)

The volume of the follicle in the formula was calculated based on a formula for the volume of a sphere and the radius of the follicle measured using ultrasonography.

In the IVG study, OCGCs were divided into the low AFC group (less than 25 follicles) and high AFC group (25 or more follicles) based on the number of antral follicles (≥2 mm in diameter) in an ovary, as described in our previous study [12]. E_2_, T, P_4_, and AMH concentrations in IVG media derived from 5 surviving OCGCs after a 12-day culture in each group were compared between groups and days of culture (days 4, 8, and 12).

## Statistical analysis

All statistical analyses were performed using software (JMP Pro 14, SAS Institute, Cary, NC, USA). All data were analyzed using a two-way analysis of variance (ANOVA). For the two-way ANOVA, we used the Fit Model platform by JMP Pro 14. The model included the effects of groups (low or high), days after follicular ablation (from days 0 to 16) or days for IVG (days 4, 8, or 12) or stages of follicular growth (selection, ovulatory, or luteal phases), and their interactions. The Student’s *t*-test or Tukey-Kramer’s honestly significant difference test were used as post-hoc tests.

## Results

### Relationships between AFC and follicular growth dynamics

**Fig. 2:**
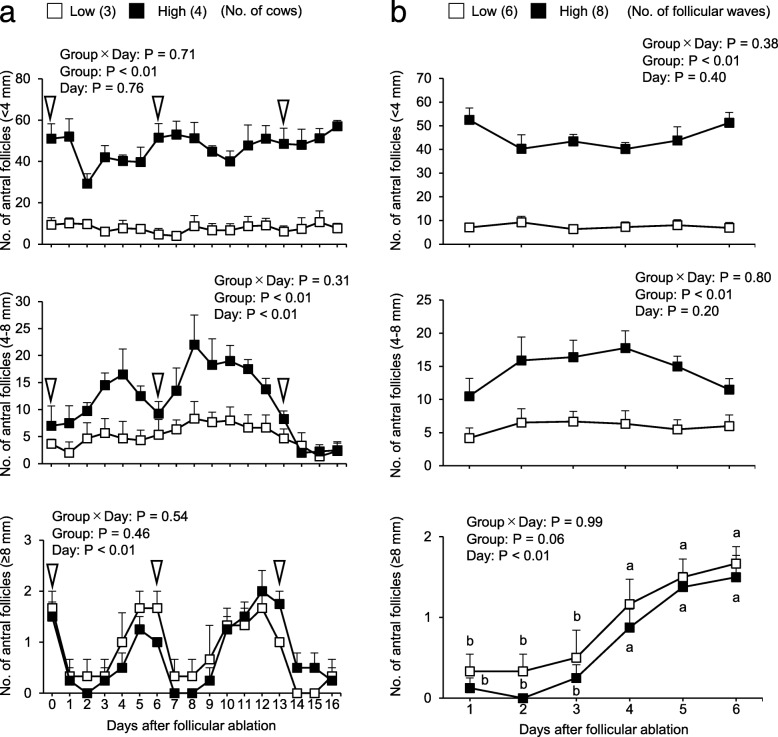
Relationship between AFC and follicular growth dynamics monitored by ultrasonography. **a**: The number of follicles after the first follicular ablation were monitored by ultrasonography. The diameters of each follicle were measured. Follicles were classified into 3 groups according to their diameters (small: < 4 mm, intermediate: 4–8 mm, and large: ≥8 mm). We compared the number of antral follicles in each category between groups and days after follicular ablation. White arrowheads indicate the timing of follicular ablation and sampling of follicular fluid. **b**: The number of small, intermediate, and large antral follicles in two follicular waves from 1 to 6 days after follicular ablation (days 0 and 6) were pooled, and we compared the number of antral follicles between groups and days after follicular ablation The results of a factorial analysis by a two-way ANOVA were shown above each panel. ^a, b^ Different letters indicate significant differences between each day (*P* < 0.05). Numbers in parentheses indicate the number of cows (**a**) or number of follicular waves (2 waves for each animal) (**b**). Error bars indicate the standard error of the mean (SEM).

The mean diameters of the largest aspirated follicles at each stage of follicular growth were similar in the low and high AFC groups. The mean total numbers of antral follicles during the experimental period from days 0 to 16 were 13.6 ± 7.6 in low AFC group (mean ± SD) and 59.2 ± 13.1 in high AFC group. As shown in Fig. 2a, the numbers of small (< 4 mm) and intermediate (4–8 mm) follicles were higher in the high AFC group than in the low AFC group (*P* < 0.01). The numbers of intermediate and large (≥8 mm) follicles changed after follicular ablation (*P* < 0.01). The numbers of small and intermediate follicles fluctuated in the high AFC group, but remained stable in the low AFC group. When the transition of the numbers of small, intermediate, and large follicles after follicular ablation was analyzed, as shown in Fig. 2b, the numbers of small and intermediate antral follicles did not show significant changes in each AFC group, whereas the number of large antral follicles increased 4 days after follicular ablation in both groups (*P* < 0.01).

### Relationship between AFC, plasma FSH, and steroid hormones

**Fig. 3:**
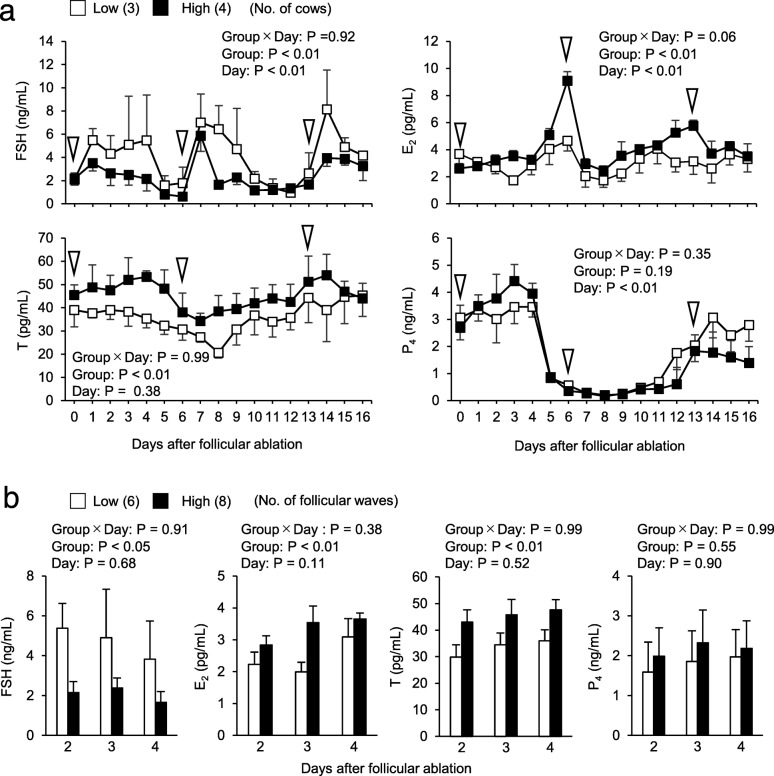
Relationship between AFC and FSH, E_2_, T, and P_4_ plasma concentrations. A: FSH and steroid hormones were measured from days 0 to 16, and we compared the plasma concentration of each hormone between groups and days after follicular ablation. White arrowheads indicate the timing of follicular ablation and sampling of follicular fluid. B: Two to four days after follicular ablation was defined as the selection phase of follicles. The selection phases in two follicular waves after follicular ablation were pooled, and we compared the plasma concentration of each hormone between groups and days after follicular ablation during that period. The results of a factorial analysis by a two-way ANOVA were shown above each panel ^a, b^ Different letters indicate significant differences between each day (*P* < 0.05). Numbers in parentheses indicate the number of cows (**a**) or number of follicular waves (2 waves for each animal) (**b**). Error bars indicate SEM.

As shown in Fig. 3a, FSH plasma concentrations were higher in the low AFC group than in the high AFC group (*P* < 0.01), while those of E_2_ and T were higher in the high AFC group than in the low AFC group (*P* < 0.01). No significant differences were observed in P_4_ plasma concentrations between the groups. Hormone plasma concentrations during the selection phase (2 to 4 days after each follicular ablation) were shown in Fig. 3b. FSH concentrations were higher in the low AFC group than in the high AFC group (*P* < 0.05), while E_2_ and T concentrations were higher in the high AFC group than in the low AFC group (*P* < 0.01).

### Relationship between AFC and steroid hormones in follicular fluid and IVG media

**Fig. 4:**
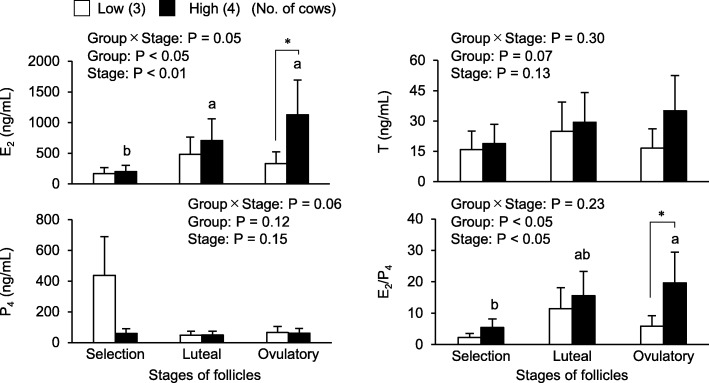
Relationship between AFC and steroidogenesis in follicular fluid. Steroid hormones in follicular fluid collected from the largest follicles in each stage of follicular growth (selection, luteal, ovulatory) were measured, and we compared the concentration of each steroid hormone and the E_2_/P_4_ ratio in follicular fluid between groups and stages of follicular growth The results of a factorial analysis by a two-way ANOVA were shown above each panel. * An asterisk indicates a significant difference between the low and high AFC groups (*P* < 0.05). ^a, b^ Different letters indicate significant differences between each stage (*P* < 0.05). Numbers in parentheses indicate the number of cows. Error bars indicate SEM.

As shown in Fig. 4, E_2_ concentrations and the E_2_/P_4_ ratio in follicular fluid were affected by AFC groups (*P* < 0.05) and the stages of follicular growth (*P* < 0.05). In the high AFC group, E_2_ concentrations in follicular fluid were higher in the luteal and ovulatory phases than in the selection phase (*P* < 0.05), while no significant difference was noted in E_2_ concentrations in follicular fluid in the low AFC group regardless of the follicular growth phase. E_2_ concentrations in follicular fluid were higher in the high AFC group than in the low AFC group at the ovulatory phase (*P* < 0.05). The E_2_/P_4_ ratio in follicular fluid was the highest in the ovulatory phase in the high AFC group, and was higher than that in the low AFC group (*P* < 0.05). T concentrations in follicular fluid were slightly higher in the high AFC group (*P* = 0.07), but were not affected by the follicular growth stage. P_4_ concentrations in follicular fluid were not affected by AFC or the follicular growth stage.

**Fig. 5:**
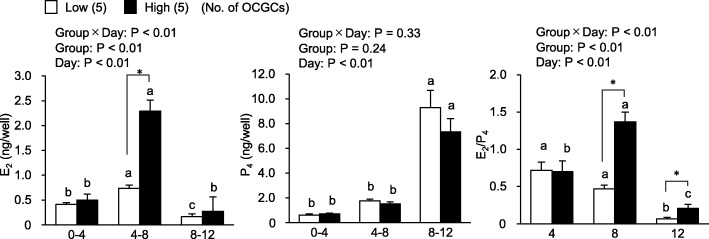
Relationships between AFC and the steroidogenesis of OCGCs during an IVG culture Steroid hormones in the IVG media of OCGCs on days 4, 8, and 12 of culture were measured, and the production of E_2_ and P_4_ and the E_2_/P_4_ ratio were calculated as described in a previous study [16]. We compared E_2_ and P_4_ concentrations and the E_2_/P_4_ ratio in IVG media between groups and the day of culture. The results of a factorial analysis by a two-way ANOVA were shown above each panel. ^a-c^: Different letters indicate significant differences between different culture periods in the same group (*P* < 0.05). * An asterisk indicates a significant difference between the low and high AFC groups (*P* < 0.05). Numbers in parentheses indicate the number of OCGCs on the same day. Error bars indicate SEM.

As shown in Fig. 5, E_2_ production from days 4 to 8 showed the highest values in all culture periods regardless of AFC, and was higher in the high AFC group than in the low AFC group (*P* < 0.05). P_4_ production increased with the extension of the culture period (*P* < 0.05), and did not significantly differ between groups. The E_2_/P_4_ ratio in the high AFC group increased from day 8 (*P* < 0.05), and was higher than that in the low AFC group (*P* < 0.05) on days 8 and 12; however, it decreased with the extension of the culture period (*P* < 0.05) in both groups.

### Relationships between AFC and AMH concentrations in plasma, follicular fluid, and IVG media

**Fig. 6:**
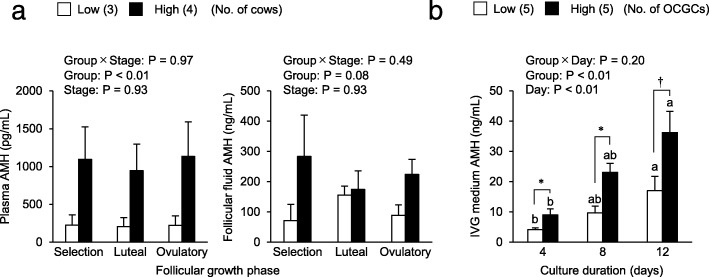
Relationships between AFC and AMH concentrations in plasma, follicular fluid, and IVG media. **a**: AMH plasma concentrations were measured on the representative days of each stage of follicular growth (selection; day 4, luteal; day 13, ovulatory; day 6). AMH concentrations in follicular fluid at each stage of follicular growth were measured using the same sample as steroid hormones. We compared AMH concentrations between groups and stages of follicular growth. **b**: AMH concentrations in IVG media of OCGCs were measured. AMH concentrations were compared between groups and days of culture (days 4, 8, and 12) The results of a factorial analysis by a two-way ANOVA were shown above each panel. * An asterisk indicates a significant difference between the low and high AFC groups (*P* < 0.05). †A dagger indicates a difference between the low and high AFC groups (*P* = 0.05). ^a, b^ Different letters indicate significant differences between each day (*P* < 0.05). Numbers in parentheses indicate the number of cows (**a**) or number of OCGCs (**b**). Error bars indicate SEM.

As shown in Fig. 6a, AMH plasma concentrations were higher in the high AFC group than in the low AFC group (*P* < 0.01) regardless of the follicular growth stage. AMH concentrations in follicular fluid were slightly higher in the high AFC group than in the low AFC group (*P* = 0.08). As shown in Fig. 6b, AMH concentrations in media increased throughout the IVG culture in each group (*P* < 0.01) and were higher in the high AFC group than in the low AFC group (*P* ≤ 0.05).

## Discussion

In our previous study [10], the normal fertilizability of oocytes was higher in the high AFC group than in the low AFC group in the 3- or 4-day interval of OPU-IVF, while this result was reversed in the 7-day interval of OPU-IVF wherein the normal fertilizability of oocytes was higher in the low AFC group than in the high AFC group. In the present study, the number of intermediate follicles increased after follicular ablation and then decreased within a few days in the high AFC group; approximately 3 to 4 days after follicular ablation, the number of intermediate follicles peaked in the high AFC group (Fig. 2a). This result indicates that most follicles 3–4 days after follicular ablation were in the growing phase in the high AFC group, resulting in the higher fertilizability of oocytes, as described in our previous study [10]. However, 7 days after follicular ablation, follicles already start to regress and oocyte fertilizability becomes low. In the low AFC group, the number of intermediate follicles was stable regardless of the number of days after follicular ablation. In the present study and a previous study [7], FSH concentrations were higher in low AFC cows than in high AFC cows. These results indicate that intermediate follicles in the low AFC group are consistently growing in the presence of a high FSH concentration, resulting in higher fertilizability in the low AFC group than in the high AFC group at the 7-day interval of OPU. The early degradation of intermediate antral follicles may be caused by higher E_2_ concentrations in the dominant follicle in the high AFC group, which may induce the degeneration of subordinate follicles [54].

In the present study, E_2_ concentrations and the E_2_/P_4_ ratio in follicular fluid at the ovulatory phase were higher in the high AFC group (1127 ng/mL) than in the low AFC group (332 ng/mL). Mossa et al. [43] also reported higher E_2_ concentrations in the dominant follicle in high AFC heifers (588 ng/m) than in low AFC heifers (435 ng/mL). A previous study using an in vitro culture of granulosa cells suggested that the lower expression levels of FSH receptors and aromatase (P450arom) resulted in impaired responses to FSH and E_2_ production by granulosa cells in low AFC cattle [41]. These findings suggest a difference in responses to a FSH stimulus between high and low AFC cattle. However, Ireland et al. [42] demonstrated that E_2_ concentrations in follicles (5–7 mm) at the emergence of the follicular wave (24 to 48 h after ovulation) were higher in low AFC heifers (approximately 90 ng/mL) than in high AFC heifers (approximately 40 ng/mL). In the present study, E_2_ concentrations in follicles of > 8 mm in diameter at the selection phase were 168 ng/mL in low AFC cows and 203 ng/mL in high AFC cows. These results indicate that the function of granulosa cells in follicles in low and high AFC cattle is altered before and after the expression of LH receptors at approximately 8 mm [49]. Furthermore, these results suggest that the ability of LH-mediated E_2_ production is impaired in the low AFC group, resulting in lower E_2_ concentrations in dominant follicles after the selection phase. Endo et al. [55] reported that E_2_ promoted the growth and maturational competence of bovine IVG oocytes. Our previous findings indicated that E_2_ production was higher by OCGCs producing matured oocytes after in vitro maturation (IVM) than by OCGCs producing immature oocytes after IVM [16]. Moreover, OCGCs derived from high AFC ovaries showed higher E_2_ production by granulosa cells and higher oocyte developmental competence than those from low AFC ovaries [12]. Consequently, impaired E_2_ production in low AFC cattle may have a negative impact on the growth, maturation, and developmental competence of oocytes, resulting in lower fertility in low AFC cattle than in high AFC cattle.

E_2_ and T concentrations were higher in the high AFC group than in the low AFC group not only in follicular fluid, but also in plasma, whereas FSH concentrations were higher in the low AFC group than in the high AFC group in the present study. Previous studies reported that T plasma concentrations were higher in the high AFC group than in the low AFC group in heifers and cows [43] and FSH plasma concentrations were higher in the low AFC group than in the high AFC group in heifers [9] and cows [7, 56]; however, E_2_ plasma concentrations were similar in low and high AFC cattle [7, 9, 56]. A possible reason for the difference in E_2_ plasma concentrations between the present and previous studies is the difference in the age of cattle used in experiments. In the present study, we used older cows (3.7, 11.4, and 14.5 years old in low AFC cows; 3.9, 4.8, 11.8, and 12.9 years old in high AFC cows) than those in previous studies (14–33 months old [9], 3–5 years old [7], and 2.6–10.8 years old [56]). In cattle, the numbers of primordial and preantral follicles are stable after birth until 4 to 6 years old and then decrease [57]. In humans, E_2_ serum concentrations begin to decrease and FSH serum concentrations markedly increase 2 years before the last menstrual period [58]. In the present study, average E_2_ plasma concentrations from days 0 to 16 were similar in low AFC cows (3.2 ± 1.5 pg/mL) and high AFC cows (3.8 ± 1.9 pg/mL) younger than 10 years old; however, they were higher in high AFC cows (4.3 ± 1.8 ng/mL) than in low AFC cows (3.0 ± 1.4 ng/mL) older than 10 years (*P* < 0.01, the Student’s *t*-test). These results indicate an age-related decrease in E_2_ plasma concentrations, particularly in low AFC cows, and that the fertility of cows decreases at younger ages in low AFC cows than in high AFC cows. We speculate that if we use only young age cows, there will be no difference in E_2_ plasma concentrations between the groups, and if we use only old age cows, E_2_ plasma concentrations will be higher in the high AFC cows. In addition to E_2_, inhibin is a major hormone causing negative feedback on FSH secretion [59]. A previous study using 3–5-year-old cows [7] suggested that inhibin-A serum concentrations were slightly higher in high AFC cows than in low AFC cows at the ovulatory phase (*P* = 0.07), but not at the selection phase of dominant follicles. Another study using 11–13-month-old heifers [42] indicated that inhibin-A concentrations in follicles (5–7 mm) at the emergence of the follicular wave (24 to 48 h after ovulation) were similar between high and low AFC heifers. Future studies are needed to investigate the relationship between AFC, age, and the competence of E_2_ and inhibin production in granulosa cells.

In the present study, AMH concentrations in follicular fluids derived from large follicles (≥8 mm) at different stages of follicular growth (selection, luteal, and ovulatory phases) were slightly higher in the high AFC group than in the low AFC group. Furthermore, AMH concentrations in the IVG media of OCGCs derived from a 4- to 12-day culture were higher in the high AFC group than in the low AFC group. Scheetz et al. [41] reported that the production of AMH and expression of the messenger ribonucleic acid of AMH were greater in cultured granulosa cells derived from high AFC cows than those from low AFC cows. These findings indicate that the ability to produce AMH by granulosa cells is higher in high AFC cows than in low AFC cows throughout follicular development. On the other hand, AMH decreased the expression of FSH receptors in human granulosa cells [60], and E_2_ production was impaired by decreasing the responses of preantral and antral follicles to FSH in mice [35], humans [36, 37], and sheep [38]. In the present study and a previous study [43], higher E_2_ concentrations in follicular fluid were observed in the high AFC group, while the AMH concentration that suppressed E_2_ secretion was higher in the high AFC group than in the low AFC group. The reason for the contradiction of AMH and E_2_ concentrations may be explained by T concentrations in follicular fluid. T has been shown to increase the transcription of FSH receptors in bovine cultured granulosa cells [61], and the in vivo results of the present study showed higher T concentrations in the high AFC group. These results suggest that higher T production by theca cells counteracts the function of AMH for reducing FSH-mediated E_2_ production in high AFC cattle. The roles of theca cells in follicular growth need to be investigated in more detail.

## Conclusions

FSH plasma concentrations were higher in low AFC cows than in high AFC cows, whereas E_2_ and T concentrations were higher in high AFC cows than in low AFC cows. These results suggest that the weaker production of E_2_ by granulosa cells in low AFC cows results in low E_2_ concentrations at the systemic level, resulting in high FSH concentrations and the consistent development of intermediate follicles in low AFC cows. Conversely, higher E_2_ concentrations suppressed FSH secretion in high AFC cows, resulting in the marked degradation of intermediate follicles at the selection phase. In vivo and in vitro AMH production by granulosa cells were higher in high AFC cows than in low AFC cows, indicating the existence of stage-dependent regulatory roles for not only AMH, but also other factors possibly derived from theca cells in FSH-mediated follicular growth and steroidogenesis in cattle.

## Data Availability

The datasets used and/or analyzed during the present study are available from the corresponding author on reasonable request.

## Abbreviations

A_4_: Androstenedione
AFC: Antral follicle count
AMH: Anti-Müllerian hormone
ANOVA: Analysis of variance
BSA: Bovine serum albumin
CMO: Carboxymethyloxime
E_2_: Estradiol-17β
FSH: Follicle-stimulating hormone
GnRH: Gonadotropin-releasing hormone
HEPES: 2-[4-(2-Hydroxyethyl)-1-piperazinyl] ethanesulfonic acid
IVF: In vitro fertilization
i.m: Intramuscular
IVG: In vitro growth
IVM: In vitro maturation
LH: luteinizing hormone
OCGC: Oocyte-cumulus-granulosa complex
OPU: Ovum pick-up
P_4_: Progesterone
P450arom: aromatase
PGF_2α_: Prostaglandin F_2α_
SD: Standard deviation
SEM: Standard error of the mean
T: Testosterone
TCM 199: Tissue culture medium 199
TMB: 3, 3′, 5, 5′-tetramethylbenzidine
